# The Effect of Micro- and Nanoscale Surface Topographies on Silk on Human Corneal Limbal Epithelial Cell Differentiation

**DOI:** 10.1038/s41598-018-37804-z

**Published:** 2019-02-06

**Authors:** Kai B. Kang, Brian D. Lawrence, X. Raymond Gao, Victor H. Guaiquil, Aihong Liu, Mark I. Rosenblatt

**Affiliations:** 1 Department of Ophthalmology and Visual Sciences, Illinois Eye and Ear Infirmary, University of Illinois at Chicago, Chicago, IL USA; 2 Department of Ophthalmology, Weill Cornell Medical College, New York, NY USA

## Abstract

We previously reported that micro- and nano-scale topographic pitch created on silk films mimic features of the corneal basement membrane by providing biophysical cues to direct corneal epithelial cell adherence and migration. However, the effect of these topographical features on corneal limbal epithelial cell differentiation has not been explored. We hypothesize in the current study that various topographical pitch created on silk may affect corneal epithelial stem cell differentiation and alter the expression of genes involved in cell differentiation and self-renewal. We patterned silk films with different topographic pitch via soft lithography and observed human corneal limbal epithelial cell behavior. Colony forming assay demonstrated increased colony forming efficiency on patterned silk films. Cells cultured on nanoscale patterned silk films also expressed lower levels of putative keratocyte differentiation markers and higher levels of putative limbal stem cell markers. RNA-Seq analysis further implicated the involvement of pathways related to stem cell differentiation and self-renewal, including Notch, ERK/MAPK and Wnt/β-catenin signaling. We conclude that patterned silk film substrates can be used as scaffolds and provide biophysical cues to corneal limbal stem cells that may maintain corneal epithelial stem cells at a less differentiated state.

## Introduction

The ocular surface can be damaged by various traumatic and chemical injuries and immune-mediated conditions. To repair corneal wounds, corneal epithelial stem cells at the limbus migrate, differentiate and proliferate. In this process, corneal epithelial stem cells depend on cues provided by the limbal stem cell niche. In addition to the biochemical features of the limbal stem cell niche, previous experiments using scanning electron microscopy (EM) have demonstrated a significant number of nanoscale topographical features in the 70 to 200 nanometer range existing in the limbal niche^1,2^. These nanoscale features have been shown to direct the ability of epithelial cells to adhere, migrate and proliferate onto the corneal epithelial basement membrane^1–7^.

Previously, our group demonstrated that silk fibroin protein can be made into highly transparent films that are suitable for ophthalmic applications^8–10^. In addition, using standard soft-lithography techniques, silk film surfaces can be easily modified to create different patterns^8,9^. This method allows for the design of various micro- and nano-scale topographical patterns on silk films to study the effect of systematic alternation of the epithelial cell microenvironment on cellular structure and function. Our previous experiments found that micro- and nano- scale patterned silk film substrates may serve as scaffolds that provide biophysical cues to epithelial stem cells. Through the process of mechanotransduction, these silk film scaffolds can alter cellular adherence and migration^9,11,12^. We also discovered that corneal limbal epithelial cells elongate along micro- and nanoscale pattern features and change epithelial cell genetic expression^9^.

Recently, experiments in epidermal stem cells suggest that changes in cell shape may affect cell differentiation^13,14^. To date, the effect of micro- and nano-scale surface topography on corneal limbal epithelial cell differentiation have not been explored. We hypothesize that micro- and nano-scale silk film topographies can change the expression of genes related to corneal epithelial cell differentiation; in addition, pathways activated by the process of mechanotransduction can potentially lead to important changes in the regulation of corneal limbal stem cell differentiation. In our current study, we utilize various silk film surface features of different pitch and width dimensions to study the response of human corneal epithelial cells when exposed to topographic cues ranging from the nano- to micro-scale. Specifically, changes in corneal limbal stem cell differentiation were observed, and then changes in gene expression were assessed. Results from the current study indicates that a variety of cellular responses related to limbal stem cell differentiation may be enhanced in the presence of surface topography on silk.

## Materials and Methods

### Production of silk films

**Figure 1:**
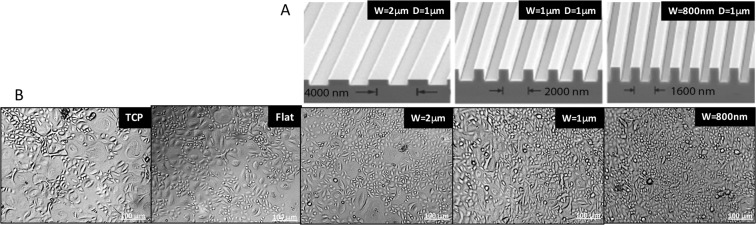
HCEC morphology. **(A**) Electron microscopy images of silicon wafers with micro- and nanoscale patterns. **(B**) Phase contrast images of primary HCECs cultured on different substrates for 14 days.

The extraction of silk solution and the production of micro- and nano- patterned silk films have been previously described^8–10,12,15^. Briefly, protein extract from Bombyx mori silkworm cocoons by (Tajima Shoji Co., Yokohama, Japan) cut in thirds and boiled in 0.02 M Na_2_CO_3_ (Sigma-Aldrich) for 40 minutes was rinsed in dH_2_O for 20-minutes and then dried overnight. This protein extract was then dissolved in 9.3 M lithium bromide at room temperature and placed in a 60 °C oven for four hours. Afterwards, the solution was dialyzed in water for 48-hours in dialysis tubing (MWCO 3,500, Pierce, Inc.) and then was centrifuged twice at 13,000-g. The resulting supernatant of aqueous silk solution with a final concentration of 8 wt./vol.% was collected and stored at 4 °C. Using standard photolithography techniques, silicon wafers with parallel ridge widths and spacing of 2 μm, 1 μm and 800 nm with 1 μm groove depths were produced (Fig. 1A)^3,4^. PDMS molds were produced from these surfaces by casting 300-mL of a 10:1 mixture of potting to catalyst solution and then cured at 60 °C for 12 hours. The patterned PDMS surfaces were then cut into 35-mm diameter casting surfaces, and then 300-μL of 8% silk fibroin solution was pipetted onto each surface. After the silk solution dried into a formed film, the cast films while still on the PDMS surfaces were placed in a chamber to allow water-anneal for up to 4-hours as previously described^8,10^. After processing, the silk films measuring 50 μm in thickness were then removed from their respective PDMS molds.

### Primary human corneal epithelial cell preparation

For preparation of primary human corneal epithelial cells (HCECs), three cornea rings of donors aged 50 to 70 were obtained with research consent and ethical approval from a regional eye bank (the Eye Bank for Sight Restoration, Inc., New York, NY). Primary cell cultures were conducted in accordance with the Association for Research in Vision and Ophthalmology Statement on Cell and Gene Therapy Research and with federal, state and local regulations. After the removal of conjunctiva, iris, corneal endothelium, and a 7-mm central corneal button, the corneoscleral ring was isolated and divided into 1 mm quadrants and placed epithelial side down on a 6-well tissue culture plate (VWR) pre-coated with 50 ug/mL of collage I (BD Biosciences, Bedford, MA, USA). These quadrants were then washed in EpiLife^**®**^ medium (Invitrogen) containing 0.1% Fungizone (Invitrogen) and 1% P/S (VWR) for 25 minutes. Each explant was then incubated overnight in 0.5 mL of EpiLife^**®**^ medium supplemented with 1% human keratinocyte growth supplement (Invitrogen), 1% P/S (VWR), 5% fetal bovine serum (FBS, Thermo Fisher Scientific Inc., Waltham, MA), 10 μg/mL mouse EGF (Invitrogen), and 100 ng/mL of cholera toxin A (Sigma-Aldrich, St. Louis, MO, USA) at 37 **°**C and 5% CO_2_. Next day, 1 mL of complete EpiLife^**®**^ medium was added. Medium was then changed every 3 to 4 days. Cell were observed daily until approximately 80% confluence was reached and then sub-cultured by digestion with TrypLE™ Express (Invitrogen) at 37 °C for 12 minutes.

Single cell suspensions at a density of 1 × 10^4^ cells/cm^2^ were seeded on tissue culture plastics plated with collagen I or silk film substrates and cultured in serum-free low-calcium medium (Epilife Medium, Invitrogen, Grand Island, NY) consisting of 10 ng/mL human recombinant EGF (Invitrogen), growth supplement, and antibiotics supplied by the manufacturer for 72 hours to 14 days. Carl Zeiss AxioCam HRm digital camera (Carl Zeiss Microimaging GmbH, Jena, Germany) was used to capture phase contrast images.

### Colony forming assay

Primary HCECs that were cultured and passaged from tissue culture plastics (TCP) were cultured onto TCP, flat silk films and silk film with feature widths of 800 nm for 14 days and then inoculated in 100-mm dishes and co-cultured with mitomycin C-treated 3T3 fibroblast cells at 5000 cells/dish for 10 to 14 days. Cells were then fixed in 4% paraformaldehyde (PFA, Electron Microscopy Sciences, Hatfield, PA, USA) and stained with Crystal Violet (Invitrogen) for 30 minutes. Colonies with at least 50 cells were counted. Colony-forming efficiency (CFE) was calculated as number of colonies per number of inoculated cells. Three independent experiments were performed.

### RNA isolation and Illumina sequencing

Our previous studies showed that patterned silk film surfaces are able to change corneal epithelial cell genetic expression at 72 hours^9^. After 72 hours of culture, HCECs were collected and Qiagen RNeasy Plus Mini Kit (Qiagen, Valencia, CA, USA) was used to extract total RNA. Agilent Technologies 2100 Bioanalyzer and Nanodrop Spectrophotometer at the Genomics Resources Core Facility (Weill Cornell Medical College, New York NY) were used to check RNA quantity and integrity.

At least 100 ng of total RNA with a minimum RNA integrity number of 8 per sample was sent to the Genomics Resources Core Facility (Weill Cornell Medical College) to perform RNA-Seq. Total RNA was used with the TruSeq mRNA-Seq Sample Preparation Kit (Illumina, San Diego, CA, USA) to construct cDNA libraries. These cDNA libraries were multiplexed with 3 samples per lane and loaded onto flow cell lanes. Sequencing-by-synthesis of 58-nucleotide length was performed on Illumina Hiseq 2000/1000 sequencing system (Illunima Inc, San Diego, CA, USA).

### Analysis of RNA-Seq data

Quality control of the RNA-Seq reads was performed using FastQC^16^ and Trimmomatic^17^. We used the maximum information quality trimmer (MAXINFO) in Trimmomatic to balance the benefits of retaining longer reads against the drawback of having low-quality bases. After QC, more than 97% reads remained. The RNA-Seq reads were aligned to the NCBI build37.2 reference genome using TopHat2^18^. The overall read mapping rate ranges 95–98%. Read count for each gene of each sample was calculated using HTSeq^19^. Differential gene expression was calculated by DESeq2^20^. Genes were considered to be differentially expressed if the absolute log-fold-change >1 and the false discovery rate <0.05. The R package^21^ was used for data visualization. Principal component analysis was performed using MATLAB (Version 9.3, The Mathworks Inc., Natick, MA).

### Pathway analysis

A data set containing differentially expressed gene identifiers and corresponding expression values was uploaded onto Ingenuity Pathways Analysis (IPA) software (Redwood, CA, USA) for pathway and network analyses. These differentially expressed gene identifiers were overlaid onto a global molecular network developed from information contained in the Ingenuity Pathway Knowledge Base, which was derived from known gene interactions published in the literature.

### Q-PCR

Using Invitrogen High Capacity RNA-to-cDNA Kit (Life Technologies, Grand Island, NY, USA), RNA isolated from cultured HCECs (200 ng per sample) was reverse transcribed into cDNA. cDNA samples were diluted 25-fold to 500 microliters. Quantitative real-time PCR (Q-PCR) was performed using Invitrogen Power SYBR Green Master Mix according to manufacturer’s protocol (Life Technologies). In this study, HCEC cultured for 72 hours and 14 days on 800 nm feature width silk film topographies were compared to HCECs cultured on flat silk and TCP controls (n = 3). The expressions of candidate genes were normalized against glyceraldehyde-3-phosphate-dehydrogenase (GAPDH). Differential expression analysis was performed using the ddCt method^22^. ANOVA (StatPlus, Version 6, AnalystSoft Inc., Walnut, CA) was used to analyze the statistical significance of Q-PCR data. Test results with *P* < 0.05 were declared statistically significant.

## Results

### Primary HCEC morphology on patterned silk films

Primary HCECs were cultured onto TCP, flat, micro- and nano-patterned silk film substrates (n = 3 per substrate). Cells were cultured for 14 days to allow cell differentiation to occur. After 14 days of culture, phase contrast images were taken to study cell morphology. Fig. 1B illustrates that primary HCECs cultured on nanopatterned silk showed the greatest homogeneity and were smaller in size in comparison to cells cultured on TCP and flat silk. Large highly differentiated epithelial cells were observed on TCP and flat silk. Cell morphology on micro- and nano-patterned silk film substrates appeared more similar in comparison to cells cultured on TCP and flat silk films.

### Primary HCEC colony forming assay

**Figure 2:**
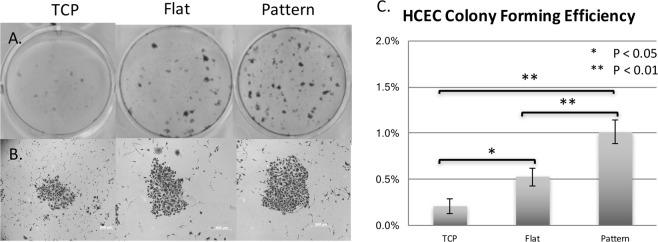
Colony forming assay. **(A**) Colony forming assay of primary HCECs after 14 days of culture on different substrates. Cells were stained with crystal violet. **(B**) Phase contrast images of a representative colony of primary HCECs cultured on different substrates. **(C**) Graph demonstrating the colony forming efficiency on different substrates. (Pattern = 800 nm film).

Figure 2A shows colony-forming assay of primary HCECs after 14 days of culture on different substrates. Colonies with at least 50 cells were counted (Fig. 2B). The average colony forming efficiency (CFE) of P2 HCECs were 0.21% on TCP, 0.55% on flat silk, and 1.0% on nanopatterned silk film. In comparison to cells cultured on TCP, primary HCECs cultured on flat silk and nanopatterned silk showed greater CFE (p = 0.02, and p = 0.001 respectively). HCECs cultured on nanopatterned silk showed greater CFE in comparison to HCECs cultured on flat silk (p = 0.002).

### Q-PCR of cell differentiation-related gene expression

**Figure 3:**
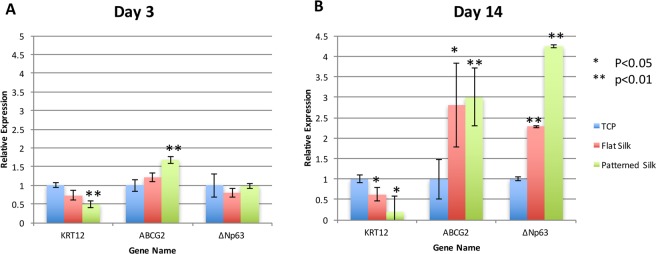
Q-PCR of cell differentiation related genes. (**A**) Q-PCR demonstrating the expression of putative cell differentiation marker KRT12, and putative corneal epithelial stem cell marker ABCG2 and ΔNp63 on different substrates at **(A**). Day 3 and **(B**). Day 14 of cultures of primary HCECs.

Quantitative real-time PCR (Q-PCR) was performed to explore levels of expression of putative limbal stem cell and keratocyte differentiation markers in primary HCECs exposed to different substrates. As shown in Fig. 3A, after 72 hours (3 days) in culture, in comparison to primary HCECs cultured on TCP, HCECs exposed to silk with nanopatterned surfaces (800 nm pattern), expressed lower levels of putative cell differentiation marker KRT12 (P = 0.001), and higher levels of putative limbal stem cell marker ABCG2 (P = 0.003). No significant changes in the levels of ΔNp63 were observed for the samples at 72 hours of culture.

Figure 3B demonstrates the expression levels of HCECs at day 14 of culture. In comparison to cells cultured on TCP, primary HCECs on flat silk and on nanoscale patterned silk expressed lower levels of putative keratocyte differentiation marker KRT12 (p < 0.05), and higher levels of putative limbal stem cell marker ABCG2 (p < 0.05) and ΔNp63 (p < 0.0001). At this time point, cells cultured on patterned silk films also expressed higher levels of ΔNp63 in comparison to cells cultured on flat silk films (p < 0.0001). Of note, in comparison to cells cultured on TCP, in cells cultured both on flat and patterned silk films, the degree of increase in the expression of putative limbal stem cell markers and decrease in the expression of keratocyte differentiation markers are greater at day 14 than at day 3.

### Sequencing run summary

**Figure 4:**
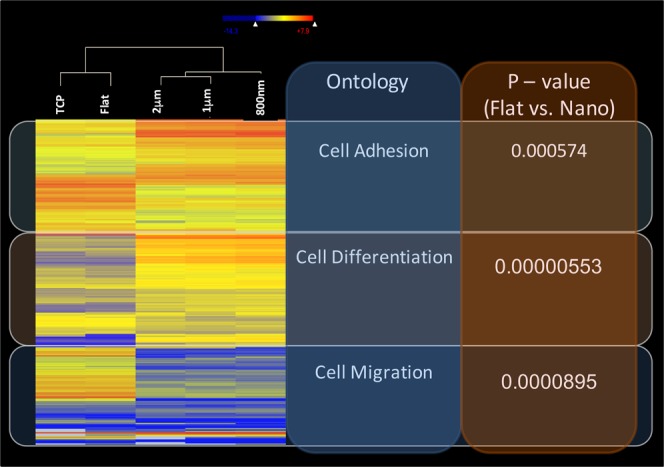
Heatmap of differentially expressed genes. Differentially expressed gene transcripts in HCECs cultured on flat silk films and on silk films with microscale (1 μm and 2 μm) nanoscale (800 nm) topographical pattern were filtered for a fold change cutoff of 1.5 and p-value cutoff of 0.05 and were used to generate gene ontology. Corresponding p-values for each gene ontological categories were shown.

To study gene expression changes more globally, fifteen libraries of primary HCEC cDNA were sequenced from cultures grown on TCP, flat silk films, and patterned silk films with parallel ridge widths of 2 μm, 1 μm and 800 nm to obtain approximately 50 to 60 million raw sequence reads per sample (n = 3). Based on our analysis workflow, 4005 differentially expressed genes with a fold change greater than 1.5 and a p-value less than 0.05 comparing flat and nano-patterned silk were found. Important ontological categories related to limbal stem cell functions were found to be significantly changed, including cell adhesion, cell migration and cell differentiation. Corresponding heat maps and p-values were shown in Fig. 4.

### Principal component analysis (PCA)

**Figure 5:**
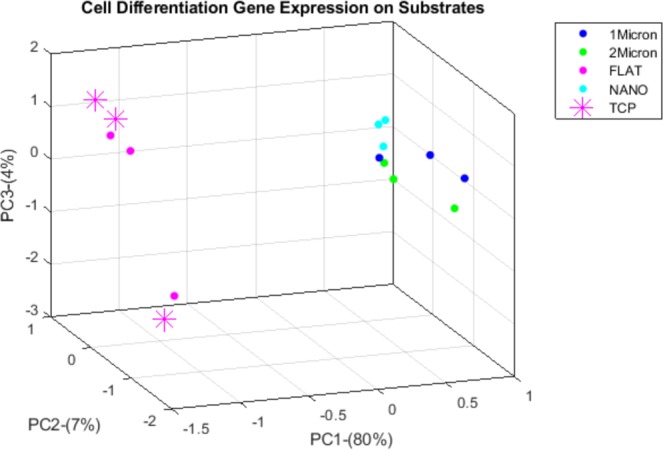
Principal component plot of cell differentiation related gene expression. Differentially expressed gene transcripts related to cell differentiation of cells cultured on TCP, flat, 1 μm, 2 μm and 800 nm silk films (3 samples from each substrates) were used to perform PCA plot of the first three principal components. Profiles group in a similar manner to the hierarchical clustering—the three patterned substrates tend to cluster together.

PCA was performed using cell differentiation related genes in our 15 samples (triplets for each experimental conditions). Fig. 5 shows a PCA plot of the first three principal components. Profiles grouped in a similar manner to the hierarchical clustering—the three patterned substrates clustered together. PCA also showed that cell differentiation pattern on the three replicates of nanopatterned silk film appeared to be the most homogenous, similar to results from our colony forming assays. In addition, it appears that the three patterned substrates clustered closely together, separating from TCP and flat silk, suggesting that cell differentiation on patterned films (in the 800 nm to 2 μm range) is distinct from cell differentiation pattern on TCP and flat silk films.

### Patterned silk films alter expression of genes involved in cell differentiation related pathways

**Figure 6:**
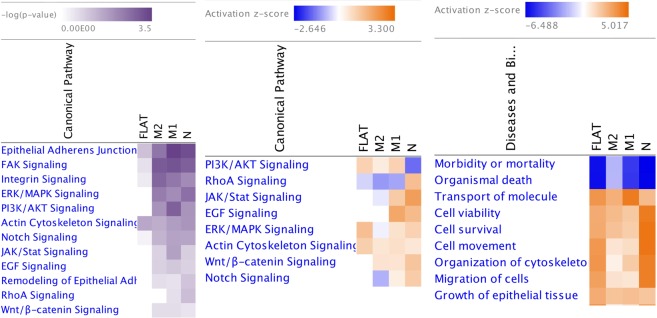
Ingenuity pathway analysis of differentially expressed genes. Differentially expressed genes for HCECs cultured on each patterned silk substrate in comparison to flat silk were analyzed using IPA. IPA analysis demonstrating the top signaling pathways and the top bio-functions involved and the corresponding p-values. (FLAT = Flat film, M2 = 2 μm film, M1 = 1 μm film, N = 800 nm film).

IPA was used to analyze the functional relationships among differentially expressed genes involved in cell differentiation. Top canonical pathways that showed significant changes according to IPKB include ERK/MAP (extracellular signal-regulated kinases/mitogen-activated protein kinases) signaling, PI3K/AKT (Phosphatidylinositol-4,5-bisphosphate 3-kinase/Protein kinase B) signaling, Notch Signaling, JAK/Stat (Janus kinase/Signal transducer and activator of transcription proteins) signaling, and Wnt (Wingless/Integrated)/β-catenin signaling (Fig. 6B). IPA analysis also demonstrates that the top bio-functions involved with positive z-scores are transport of molecules, cell viability, cell survival, cell movement, migration of cells and growth of epithelial tissue. Bio-functions with negative z-scares are morbidity of mortality and organismal death suggesting that these bio-functions were decreased on flat and patterned silk films in comparison to TCP.

### Q-PCR revealed differentially expressed genes involved in Notch signaling

**Figure 7:**
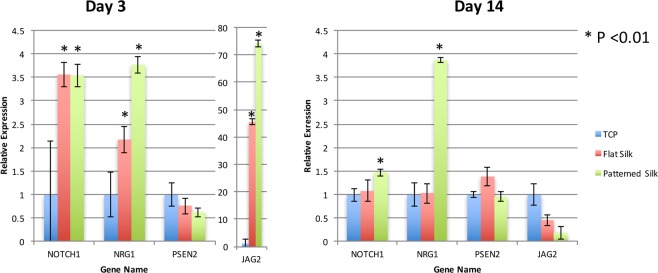
Q-PCR verification of Notch signaling pathway. Q-PCR verification illustrating fold change of Notch signaling pathway genes on TCP, flat silk film and nano-patterned silk film after 3 days **(A)** and 14 days **(B)** of culture.

The RNA-Seq results were confirmed by Q-PCR of select genes related to Notch signaling pathway. As shown in Fig. 7A, after three days in culture, primary HCECs exposed to silk with nanopatterned surfaces (800 nm pattern), expressed higher levels of Notch1, NRG, and Jag2 in comparison to HCECs cultured on flat silk films (p < 0.001). Primary HCECs were then cultured for 14 days on silk film substrates to examine the long term gene expression of cells exposed to nano-patterned surfaces (Fig. 7B). Q-PCR showed increased expression Notch1, NRG1 on patterned silk (p < 0.01).

The results of Q-PCR were compared to fold changes found in our RNA-Seq analysis. It was found that good correlation (R^2^ = 0.60) exists between the two analyses.

## Discussion

In this study, it was demonstrated that micro- to nanoscale surface topographies in the range of 800 nm to 2 μm on silk films provide biophysical cues that alter HCEC morphology and differentiation and can lead to changes in cell differentiation-mediated gene expression and activate related cell signaling pathways. It appears that corneal limbal epithelial cells cultured on nano- to microscale surface topographies are more likely to maintain stem cell like properties and morphologies. This study explored the ability of systematically modifying surface topographies on silk in the alteration of limbal stem cell differentiation with the primary intent of engineering a biomaterial suitable to use in ocular surface regeneration.

Previously, it was demonstrated that altering cell environmental conditions could effectively alter the differentiation state of stem cell populations^23^. We hypothesized that micro- to nanoscale surface topographies may be effective in expanding limbal epithelial progenitor cells. As expected, culturing primary HCECs on nanoscale patterned silk films produced cells with higher colony forming efficiency. Furthermore, after 14 days of culture, limbal stem cells on silk films with micro- and nanoscale surface features showed more homogeneity in morphology and were smaller in cell size in comparison to cells cultured on TCP. On TCP, cells were heterogeneous in morphology, representing epithelial cells at different stages of differentiation. In addition, we looked at gene expression patterns of HCEC cultures on different silk substrates both in the short term (at 72 hour of culture) and the long term (at 14 days of culture). HCECs on nanoscale patterned silk expressed lower levels of putative keratocyte differentiation marker KRT12 and higher levels of putative limbal stem cell marker ABCG2 and ΔNp63. These results demonstrate that nanoscale surface topographies on silk can maintain HCECs in a more undifferentiated, or more stem cell-like state.

The mechanisms involved in the maintenance of progenitors on silk film with nanoscale patterns are still under investigation. It was previously shown that the limbal stem cells niche has unique biochemical and physical properties that maintains limbal stem cells at a more stem cell-like state. Different studies have shown that signals such as Wnt/β-catenin pathway, Sonic hedgehog (Shh) pathway, TGF-β/BMP, and Notch pathway play important roles in the control of different types of stem cell niche^24,25^. Conditional inactivation of various key molecules involved in these signaling pathways, e.g. Notch signaling pathway, in adult mice can lead to keratinization and hyperplasia of the corneal epithelium, mimicking epidermal differentiation^26^. In our study, RNA-Seq analysis of cell differentiation-related gene expression revealed that surface topographies on silk can lead to changes in the expression of genes involved in signaling pathways previously implicated in the maintenance of the stem cell niche, including canonical Wnt receptor signaling pathway, ERK/MAPK signaling pathway, Notch signaling pathway, etc. Previous studies also suggested a “mechanoforce model” for the activation of various signaling pathways, e.g. Notch signaling^27^. It was suggested that mechanical force can facilitate the substantial conformational changes deemed necessary to uncover the cleavage site required to activate Notch receptor^27^.

**Figure 8:**
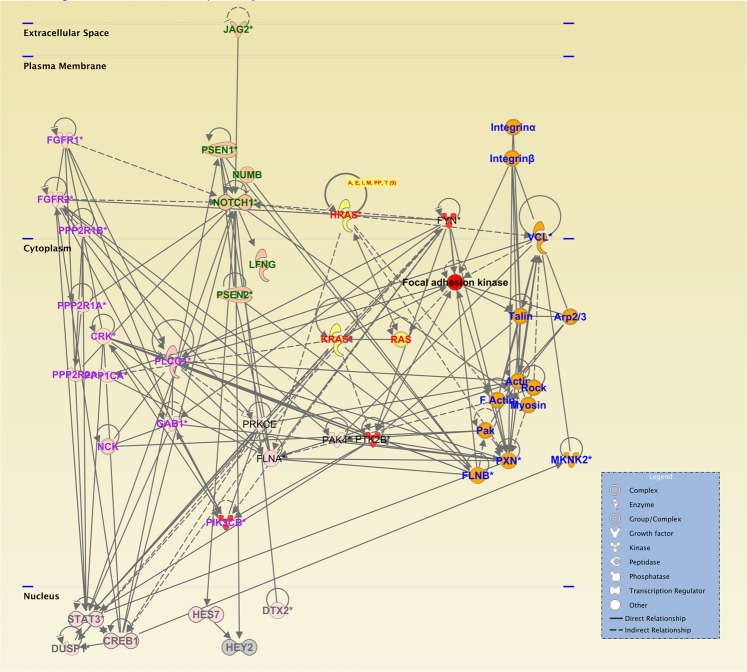
IPKB analysis of relationships among upregulated genes on silk film topography. (1) Cell surface integrin receptors sense biophysical cues provided by surface topographical patterns on silk (blue texts) which leads the the activation of key regulators of actin remodeling and recruitment of focal adhesion kinase (dark black text). (2) FAK then induces the activation of RAS superfamily of small GTPases (red texts), (3) leading to the activation of NOTCH signaling pathway (green texts) and (4) ERK/MAPK (extracellular signal-regulated kinases/mitogen-activated protein kinases) signaling pathway (purple texts). (5) The activation of these pathways leads to transcriptional changes (grey texts) related to cell proliferation and differentiation.

Our proposed pathway of mechanotransduction by nano- to micro-scale topographies on silk is demonstrated by our IPKB analysis of relationships among upregulated genes on nanoscale patterned silk films (Fig. 8). Previously, our analyses proposed that cell surface integrin receptors sense biophysical cues provided by topographical patterns on silk^9^. The clustering and activation of integrin receptor can activate key regulators of actin remodeling and recruitment of focal adhesion kinase (FAK), which then induces the activation of RAS superfamily of small GTPases. These GTPases participates in the regulation of Notch and ERK/MAPK signaling pathways, which are involved in transcriptional changes related to cell proliferation and differentiation. Our analyses demonstrates it is possible that physical properties and force exerted by surface topographies can lead to activation of various signaling pathways involved in the maintenance of limbal stem cells.

We have demonstrated that transparent silk films can serve as modifiable biomaterials for use in ophthalmic related applications. Limbal epithelial progenitor cells also can be efficiently expanded on silk films with nano-to microscale surface topographies. Currently, the management of patients with limbal stem cell deficiency remains challenging for the clinicians. Maintaining limbal progenitor cells in a less differentiated state on silk films allows for the engineering of transplantable corneal epithelial sheets and may offer another promising option for patients with limbal stem cell deficiency.

## Data Availability

The datasets generated during and/or analyzed during the current study are available from the corresponding author on reasonable request.
